# Peculiarities of the initial psychopathological manifestations after the transferred coronavirus disease COVID-19

**DOI:** 10.1192/j.eurpsy.2023.1697

**Published:** 2023-07-19

**Authors:** N. O. Maruta, V. Y. Fedchenko, I. O. Yavdak, O. R. Lapinska

**Affiliations:** 1 Borderline psychiatry, “Institute of Neurology, Psychiatry and Narcology of NAMS of Ukraine” SI, Kharkiv, Ukraine

## Abstract

**Introduction:**

The pathogenesis of mental disorders occurring during the coronavirus pandemic 2 (SARS-COV-2) includes biological and psychosocial factors. Psychopathological consequences associated with the coronavirus disease COVID-19 may occur in different groups of individuals, including patients with a history of COVID-19 and patients with psychiatric disorders preceding COVID-19.

**Objectives:**

To investigate the peculiarities of the initial psychopathological manifestations in patients with newly diagnosed mental disorders who suffered from COVID-19 and were exposed to the stressors of the SARS-CoV-2 pandemic.

**Methods:**

The study involved 97 patients with newly diagnosed mental disorders who suffered from COVID-19 and were exposed to the stressors of the SARS-CoV-2 pandemic (F 32.0-32.2 – 34 patients, F 40-45 – 32 patients, F 06.3-06.6 – 31 patient). The average age of the examined group was 44.82 ± 5.64 years. Clinico-psychopathological, psychodiagnostic, statistical methods were used.

**Results:**

In the structure of initial psychopathological manifestations detected in patients, the following prevailed: with depressive episodes - weakness and fatigue / asthenia ((87.50 ± 5.94) %), low mood / depression ((71.88 ± 8.07) %) and sleep disturbances ((65.63 ± 8.53) %); with neurotic, stress-related and somatoform disorders – weakness and fatigue / asthenia ((72.73 ± 7.87) %), decreased concentration of attention, memory / cognitive disorders ((66.67 ± 8.33 ) %) and feeling of inner tension ((60.61 ± 8.64) %); with mental disorders of organic genesis – a feeling of internal tension ((75.00 ± 7.78) %), anxiety ((62.50 ± 8.70) %) and weakness and fatigue / asthenia ((59.38 ± 8 .82) %).

In patients with depressive episodes, initial psychopathological manifestations in the form of asthenia, low mood, cognitive disorders, and sleep disorders were detected in a significantly greater number of cases compared to patients with mental disorders of organic genesis (р < 0,05). In the examined subjects of this subgroup, initial psychopathological manifestations in the form of a decrease in mood were recorded in a significantly greater number of cases compared to patients with neurotic, stress-related and somatoform disorders (р < 0,05). In patients with neurotic, stress-related and somatoform disorders, initial psychopathological manifestations in the form of anxiety, fears and obsessions were noted in a significantly smaller number of cases compared to those examined with mental disorders of organic genesis (р < 0,05).

**Conclusions:**

The obtained data will make it possible to improve the effectiveness of diagnosis and therapy of mental disorders, the formation, course and clinical picture of which were affected by the coronavirus disease COVID-19.

**Disclosure of Interest:**

None Declared

